# Comparative clinical features and outcomes of invasive pulmonary aspergillosis following influenza *versus COVID-*19: a retrospective cohort study

**DOI:** 10.3389/fmed.2026.1790007

**Published:** 2026-04-24

**Authors:** Renwen Zhang, Jun Liu, Ran Li, Li Gu

**Affiliations:** Department of Infectious Diseases and Clinical Microbiology, Beijing Institute of Respiratory Medicine and Beijing Chao-Yang Hospital, Capital Medical University, Beijing, China

**Keywords:** CAPA, COVID-19, IAPA, influenza, invasive pulmonary aspergillosis, prognosis

## Abstract

**Background and aim:**

Patients with influenza or Coronavirus disease 2019 (COVID-19) are at risk of developing invasive pulmonary aspergillosis (IPA). Whether the clinical profiles and outcomes differ between influenza-associated pulmonary aspergillosis (IAPA) and COVID-19-associated pulmonary aspergillosis (CAPA) remains unclear, hindering efforts to optimize management. This study aimed to compare the demographic, clinical, laboratory characteristics, and outcomes of patients with IPA following influenza A/B versus COVID-19 during the same period.

**Methods:**

We conducted a single-center retrospective cohort study in China from December 1, 2022, to September 1, 2024. The study included 45 patients with IAPA and 82 patients with CAPA. We compared demographics, clinical features, management, and mortality between the IAPA and CAPA patients. Group comparisons utilized Student’s *t*-test or the Mann-Whitney *U* test for continuous variables, the Chi-square or Fisher’s exact test for categorical variables, and Kaplan-Meier survival curves, and time-dependent Cox proportional hazards model for survival data.

**Results:**

Compared to IAPA patients, CAPA patients had significantly lower lymphocytes, especially in CD4^+^ T cells, CD8^+^ T cells, and B cells (all *p* < 0.05). Corticosteroid use was more frequent among CAPA patients than IAPA patients. The median time from viral diagnosis to IPA detection was longer in CAPA patients than in IAPA patients. Respiratory co-infections (bacterial) were more common in the CAPA group (*p* = 0.030). After adjusting for confounders, the risk of death within the first 14 days following IPA diagnosis was 4.92 times higher in the CAPA group than in the IAPA group (HR = 4.92, 95% CI: 1.35–18.01, *p* = 0.016).

**Conclusion:**

CAPA was independently associated with an approximately five-fold increase in the risk of death within the first 14 days following IPA diagnosis. This early hazard, together with the higher frequency of corticosteroid use, respiratory co-infections (bacterial), and severe lymphopenia, underscores a critical window for early therapeutic intervention in patients with CAPA.

## Introduction

Influenza and COVID-19 are major causes of severe viral pneumonia. They impose a substantial burden on global health systems (1, 2). Both viruses can induce profound pulmonary inflammation and immune dysregulation, increasing susceptibility to respiratory co-infections (3–5). Notably, invasive pulmonary aspergillosis (IPA) has emerged as a critical and lethal complication (6, 7).

Influenza-associated pulmonary aspergillosis (IAPA) is well-recognized, affecting approximately 16–28% of critically ill influenza patients in the intensive care unit (ICU) and contributing to high mortality (8–10). Similarly, during the COVID-19 pandemic, COVID-19-associated pulmonary aspergillosis (CAPA) was frequently identified as a leading fungal infection among severely ill patients, significantly worsening clinical outcomes (11–16). Both IAPA and CAPA highlight the interplay between viral lung injury and invasive fungal disease. However, it remains unclear whether the clinical presentation, risk factors, disease course, and outcomes of IPA differ substantially depending on the preceding viral pathogen. The host immune response, including the degree of lymphopenia and the therapeutic use of immunomodulators like corticosteroids, differs between influenza and COVID-19. This variation may in turn shape the phenotype of subsequent IPA (7, 17–19). Understanding these distinctions is critical for tailoring surveillance, diagnostic timing, and management strategies.

To date, direct comparative studies of IAPA and CAPA remain scarce. Existing research often conducted in different epidemiological periods, suggests potential differences in their clinical presentation, such as the degree of immunosuppression and the timing of aspergillosis onset (20–24). However, these studies have predominantly focused on risk factors for development, with limited concurrent, head-to-head comparison of clinical features and, more importantly, factors associated with mortality within the same timeframe (20–26). Therefore, we conducted a single-center retrospective cohort study. Our goal was to directly compare the demographic, clinical, laboratory, and outcome characteristics of patients with IPA following influenza A/B versus COVID-19 during the same period. We aimed to identify pathogen-specific patterns to inform more precise clinical management.

## Materials and methods

### Ethics approval

This retrospective study received ethical approval from the institutional review board of ethics committee at Beijing Chaoyang Hospital, affiliated with Capital Medical University (Approval No.: 2026-KE-10) and was conducted in alignment with the Declaration of Helsinki (1964) and its subsequent amendments or comparable ethical standards. Given the retrospective nature of the study, which involved the analysis of anonymized clinical data, the ethics committee formally waived the requirement for informed consent.

### Study participants

We performed a single-center retrospective cohort study involving consecutive patients admitted to Beijing Chao-Yang Hospital, Capital Medical University, from December 1, 2022, to September 1, 2024. Our study adhered to the STROBE guidelines.

Inclusion criteria: (1) a positive polymerase chain reaction (PCR) test for severe acute respiratory syndrome coronavirus 2 (SARS-CoV-2) or influenza A/B obtained from nasopharyngeal or throat swabs; (2) evidence of lower respiratory tract infection (fever, dyspnea, and radiographic lung infiltrates); (3) fulfillment of the diagnostic criteria for invasive pulmonary aspergillosis (IPA) as defined by the European Organization for Research and Treatment of Cancer/Mycoses Study Group (EORTC/MSG) (25); (4) invasive pulmonary aspergillosis following influenza or COVID-19.

Exclusion criteria: (1) individuals aged less than 18 years; (2) patients with pre-existing *Aspergillus* infection or IPA before influenza or COVID-19; (3) incomplete clinical data.

All respiratory samples were collected under aseptic conditions and processed immediately in the microbiology laboratory. Culture positivity was confirmed by standard mycological techniques, and growth of *Aspergillus* was considered significant only when accompanied by compatible clinical and (where available) radiological findings.

### Clinical data collection

Data were retrospectively extracted from electronic medical records (EMRs) and included: baseline characteristics (age, sex, body weight), comorbidities (Arterial hypertension, Diabetes mellitus, Coronary heart disease, cancer, etc.), clinical course and interventions (length of hospital stay, ICU admission, oxygen therapy requirements, utilization of mechanical ventilation (MV), continuous renal replacement therapy (CRRT), etc.), as well as microbiological and laboratory data (results from galactomannan (GM) tests, fungal cultures, and other pertinent laboratory parameters). All data were obtained from the hospital’s EMR system. Diagnostic assessments (PCR, GM assays, cultures, microscopy) were performed as part of standard clinical care in accordance with established hospital protocols.

### Diagnostic criteria

In our study, the diagnostic criteria were as follows: IAPA utilized the IAPA expert consensus criteria by Verweij and colleagues (5), while CAPA adopted the CAPA criteria established by the European Confederation of Medical Mycology (ECMM) and the International Society for Human and Animal Mycology (ISHAM) (26). It is important to note that histopathological examination was not included in this investigation, which may have constrained the pathological confirmation of IPA. The patients with probable IAPA and CAPA included in the study all had imaging evidence of pulmonary infiltrates, but this was not limited to chest CT; due to the severity of their illness, some patients only had chest X-ray results available.

Probable IAPA is defined based on recent expert consensus (5), necessitating the presence of pulmonary infiltrates along with at least one of the following: a serum GM index greater than 0.5, a bronchoalveolar lavage fluid (BALF) GM index over 1.0, or a positive *Aspergillus* culture from BALF. Alternatively, a cavitating infiltrate that cannot be attributed to another etiology, along with a positive culture from sputum or BALF, also qualifies as probable IAPA.

Probable CAPA: this was defined according to the modified 2020 ECMM/ISHAM consensus criteria (26). The presence of pulmonary infiltrates, which may or may not exhibit cavitation, coupled with clinical worsening and mycological indicators-such as a positive culture from BALF, a serum GM index exceeding 0.5, or a BALF GM index greater than 1.0-constitutes a diagnostic criterion for IPA.

Respiratory co-infection is usually defined as the presence of two or more different pathogens (including viruses, bacteria, fungi, and atypical pathogens) detected simultaneously or sequentially within a short period in the same acute respiratory infection event occurring in an individual, using reliable molecular diagnostic techniques (27–32). In this study, respiratory co-infection was defined by the identification of a clinically significant bacterial or fungal pathogen in BALF, sputum, or blood culture, prompting the initiation of targeted antibiotic or antifungal therapy, respectively.

Respiratory failure (33) was defined based on admission criteria according to the following: (1) partial pressure of oxygen (PaO_2_) < 60 mmHg or oxygen saturation (SpO_2_) < 90% on room air, or (2) PaO_2_/fraction of inspired oxygen (FiO_2_) ratio ≤ 300 mmHg, as assessed upon hospital admission.

### Statistical analysis

Statistical analysis was conducted where categorical variables were presented as counts and percentages, with comparisons made using the Chi-square test or Fisher’s exact test, as appropriate. Continuous variables were reported as mean ± *standard deviation* (SD) if they followed a normal distribution and compared using independent samples *t*-tests. Non-normally distributed data were expressed as medians with interquartile ranges (IQR, 25–75%) and assessed using the Mann-Whitney *U* test. Kaplan-Meier survival curves for various subgroups were constructed and analyzed using the log-rank test. In the Kaplan-Meier survival curves “day 0” on the x-axis is defined as the date of IPA diagnosis. To address potential violations of the proportional hazard assumption over time, time-dependent Cox proportional hazards model was used to evaluate the effect of exposure on the outcome. The model was adjusted for covariates including age, sex, lymphocytes, chronic pulmonary disease, and immunocompromised status. The proportional hazards assumption was assessed using tests based on Schoenfeld residuals. All statistical tests were two-sided, with a *p*-value of less than 0.05 deemed statistically significant. The data analysis was executed using Free Statistics software (version 1.9.2).

## Results

**FIGURE 1:**
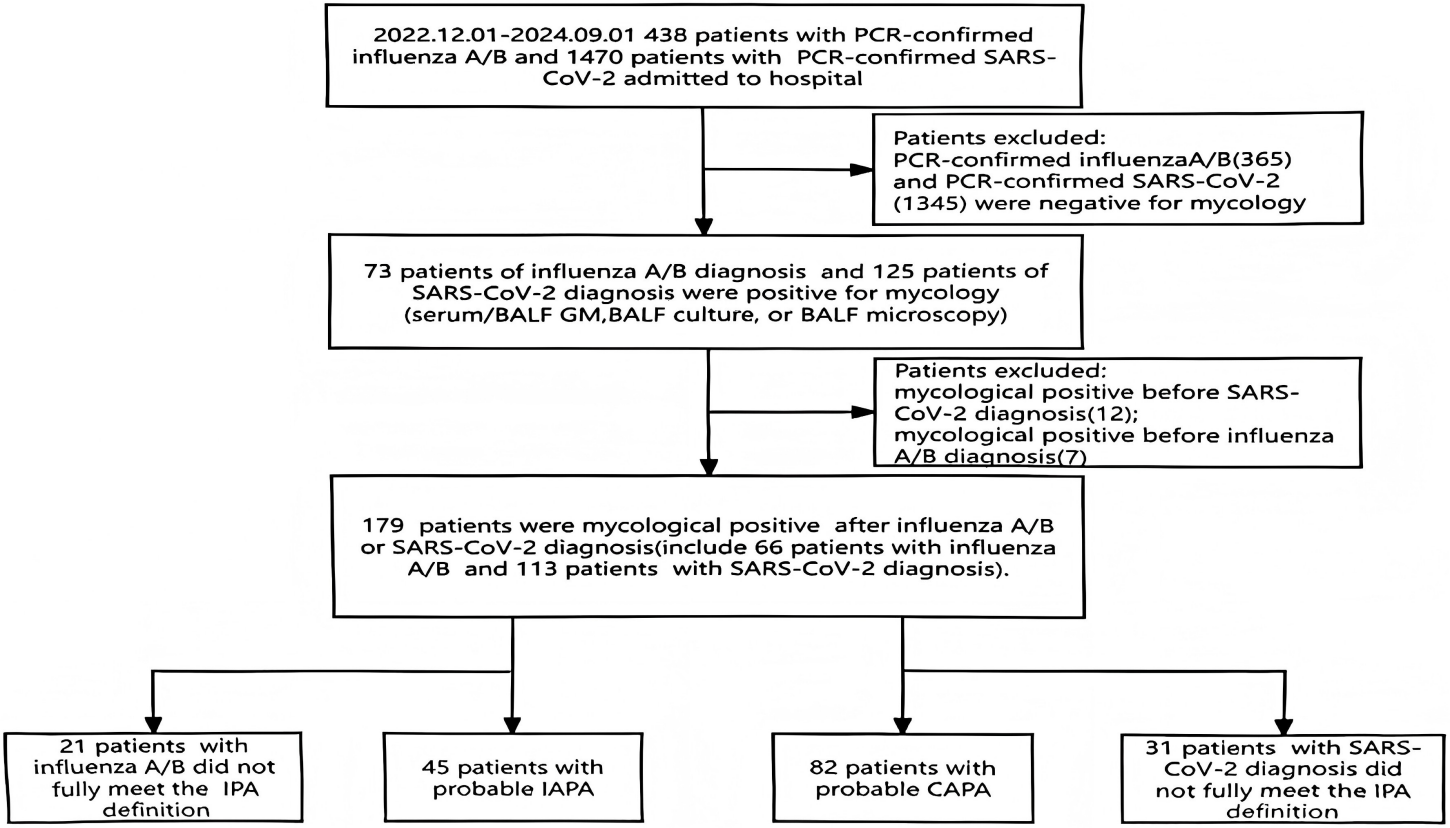
Flowchart of the study including patients. IPA: invasive pulmonary aspergillosis; IAPA: influenza- associated invasive pulmonary aspergillosis; CAPA: COVID-19-associated invasive pulmonary aspergillosis; GM: galactomannan; BALF:bronchoalveolar lavage fluid.

In total, this study evaluated 438 patients who tested positive for influenza virus nucleic acid and 1470 patients who tested positive for coronavirus nucleic acid. Among these, the positivity rates for IPA were 73 out of 438 (16.7%) and 125 out of 1470 (8.5%), respectively, revealing a statistically significant difference in incidence rates between the two groups (*p* < 0.001). Following the inclusion and exclusion criteria of this study, a total of 127 patients were enrolled, comprising 45 IAPA patients and 82 CAPA patients (see in Figure 1). Details regarding the baseline immune status of the study population are available in Supplementary material. In this cohort, no patients received antifungal prophylaxis prior to diagnostic sampling. Empirical antifungal therapy was initiated only after sampling in the vast majority of cases.

### Clinical characteristics and test results

The clinical characteristics, demographic data, and underlying conditions of the patients are summarized in Table 1. A significant age difference (*p* = 0.012) was noted between IAPA patients (62.4 ± 16.5 years) and CAPA patients (69.0 ± 12.5 years). No notable difference was found between the two groups concerning other clinical characteristics. Among the underlying conditions, CAPA patients exhibited a significantly higher prevalence of chronic kidney disease (*p* = 0.028) and chronic liver disease (*p* = 0.032). Conversely, the proportion of patients with chronic pulmonary disease was significantly lower (*p* < 0.001) in the CAPA cohort (35.4%) compared to the IAPA cohort (68.5%). Additionally, the lymphocytes were also assessed. The comparison of immune cell counts, specifically CD4^+^T cells, CD8^+^T cells, and B cells, revealed significant differences between patients with CAPA and IAPA, as outlined in Table 1. Notably, CAPA patients exhibited lower immune cell counts. Additionally, fever and diarrhea were observed to be more prevalent among CAPA patients, with *p*-values of 0.015 and 0.019, respectively. Sensitivity results for patients after excluding individuals with prior immunosuppression are detailed in Supplementary Table S1.

**TABLE 1:** Baseline features of the study patients.

Variables	Total (*n* = 127)	CAPA patients (*n* = 82)	IAPA patients (*n* = 45)	*p*-value
Demographics				
Male, n (%)	84 (66.1)	59 (72.0)	25 (55.6)	0.062
Age, *Mean* ± *SD*, years	66.7 ± 14.4	69.0 ± 12.5	62.4 ± 16.5	**0.012**
BMI, *Mean* ± *SD*, kg/m^2^	23.3 ± 3.8	23.3 ± 4.2	23.3 ± 3.2	0.982
Underlying conditions, n (%)				
Arterial Hypertension	64 (50.4)	42 (51.2)	22 (48.9)	0.802
Coronary artery disease	59 (46.5)	43 (52.4)	16 (35.6)	0.068
Cerebrovascular disease	13 (10.2)	7 (8.5)	6 (13.3)	0.542
Diabetes mellitus	50 (39.4)	30 (36.6)	20 (44.4)	0.386
Chronic kidney disease	17 (13.4)	15 (18.3)	2 (4.4)	**0.028**
Chronic pulmonary disease	60 (47.2)	29 (35.4)	31 (68.9)	**< 0.001**
Chronic liver disease	13 (10.2)	12 (14.6)	1 (2.2)	**0.032**
Hematological malignancy	9 (7.1)	7 (8.5)	2 (4.4)	0.490
Solid organ transplantation	7 (5.5)	6 (7.3)	1 (2.2)	0.420
Autoimmune disease	15 (11.8)	7 (8.5)	8 (17.8)	0.123
Solid organ malignancy	11 (8.7)	6 (7.3)	5 (11.1)	0.518
Thrombosis	18 (14.2)	13 (15.9)	5 (11.1)	0.464
Vaccine, n (%)	29(22.8)	17(20.7)	12(26.7)	0.446
Immunocompromised status	17(13.4)	13(15.9)	4(8.9)	0.270
Symptoms, n(%)				
Fever	111 (87.4)	76 (92.7)	35 (77.8)	**0.015**
Cough	115 (90.6)	73 (89.0)	42 (93.3)	0.537
Hemoptysis	22 (17.3)	13 (15.9)	9 (20.0)	0.555
Diarrhea	14 (11.0)	13 (15.9)	1 (2.2)	**0.019**
Vomit	8 (6.3)	7 (8.5)	1 (2.2)	0.258
Sore throat	20 (15.7)	13 (15.9)	7 (15.6)	0.965
Laboratory tests at admission				
Leukocyte count, median (IQR), × 10^9^/L	8.6(5.7, 11.8)	9.0(5.6, 11.8)	7.9(6.1, 10.8)	0.692
Neutrophils, median (IQR), × 10^9^/L	6.7(4.5, 10.4)	7.5(4.6, 10.7)	6.2(4.4, 9.3)	0.426
Lymphocytes, median (IQR), × 10^9^/L	0.6(0.3, 1.2)	0.5(0.3, 0.9)	0.8(0.6, 1.6)	**< 0.001**
Hemoglobin, median (IQR), g/L	121.0 ± 22.1	119.4 ± 24.0	124.0 ± 17.9	0.269
CRP, median (IQR), mg/L	50.4(18.7, 107.0)	55.0(13.4, 107.8)	48.0(23.0, 103.0)	0.579
Creatinine, median (IQR), μmol/L	62.3(51.5, 87.1)	64.2(52.4, 88.1)	59.5(48.5, 80.0)	0.400
PCT, median (IQR), ng/mL	0.2(0, 0.9)	0.2(0, 1.0)	0.1(0, 0.8)	0.945
CD4^+^Tcells, median (IQR), cell/μL	172.0(77.0,346.5)	138.5(69.8,262.)	257.0(137.0,571.0)	**0.001**
CD8^+^ T cells, median (IQR), cell/μL	108.0(56.0,252.5)	101.0(41.2,152.0)	183.0(93.0, 392.0)	**0.001**
NK cells, median (IQR), cell/μL	59.0(30.5, 138.0)	53.5(29.0, 123.2)	77.0(35.0, 212.0)	0.129
B cells,median (IQR), cell/μL	76.0(18.5, 146.5)	49.0(17.0, 129.0)	98.0(27.0, 164.0)	**0.049**
D- -Dimer, median (IQR), ng/L	1.2(0.6, 2.7)	1.2(0.8, 3.0)	1.0(0.5, 2.0)	0.209

Data are presented as number (%) or median (IQR) unless otherwise indicated. Bold indicated data with a significant difference. BMI, body mass index; CRP, C-reaction protein; PCT, procalcitonin; NK, natural killer cell; MV, mechanical ventilation.

### Laboratory diagnostics and distribution of invasive pulmonary aspergillosis

In terms of laboratory diagnostics for IPA, the methods employed included BALF and sputum cultures. In our study, there were cases of repeated sample submissions; therefore, only the results of the first culture from each patient were included in the statistical analysis (for both BALF and sputum), and presented in Table 2. The positivity rates of the GM tests were 40.0% in blood serum and 76.2% in BALF. In the CAPA group, 35 cases underwent both sputum culture and BALF culture, while in the IAPA group, 16 cases underwent both sputum culture and BALF culture. Comparatively, patients with IAPA demonstrated a higher sputum culture positivity rate than those with CAPA. However, the positivity rates of GM tests in both serum and BALF did not show significant differences between CAPA and IAPA patients. Sensitivity results for patients after excluding individuals with prior immunosuppression are detailed in Supplementary Table S2.

**TABLE 2:** Laboratory diagnositics of *Aspergillus* of the 127 enrolled patients.

Variables	Total (*n* = 127)	CAPA patients (*n* = 82)	IAPA patients (*n* = 45)	*p*-value
BALF culture, n	96	64	32	
BALF culture (+), n (%)	71(74.0)	45(70.3)	26(81.3)	0.250
Sputum culture, n	60	37	23	
Sputum culture (+), n (%)	16(26.7)	6(16.2)	10(43.5)	**0.020**
Mircoscopy, n	55	40	15	
Mircoscopy (+), n (%)	12(21.8)	6(15.0)	6(40.0)	**0.046**
Serum GM, n	115	80	35	
Serum GM (+), n (%)	46(40.0)	32(40.0)	14(40.0)	1.000
BALF GM, n	42	26	16	
BALF GM (+), n (%)	32(76.2)	22(84.6)	10(62.5)	0.102

Data are presented as number (%). Bold indicated data with a significant difference. There were cases of repeated sample submissions per patient; however, to maintain statistical independence and avoid inflation of the incidence rate, only the results of the first culture from each patient were included in the final statistical analysis.

**FIGURE 2:**
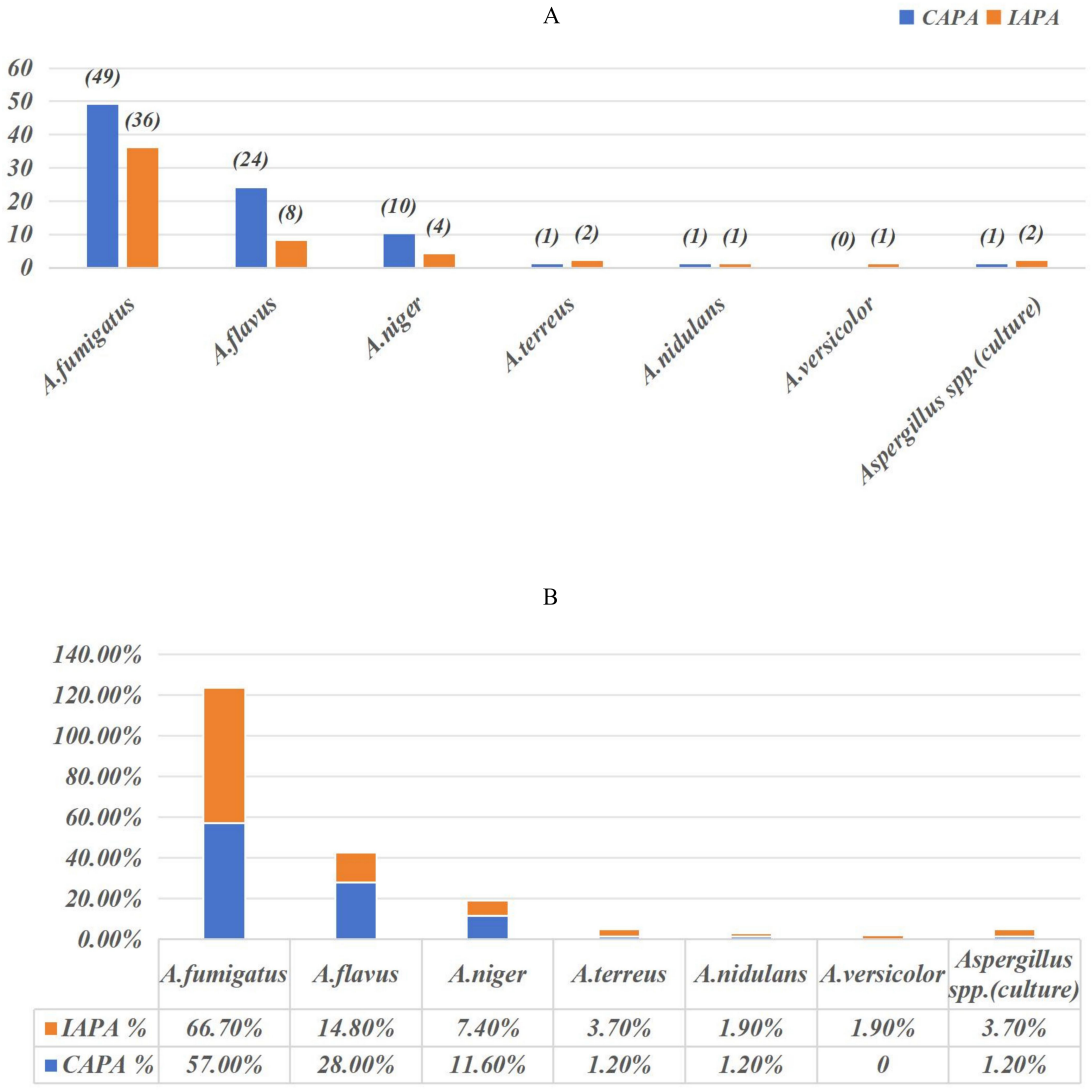
Aspergillus isolated from the 127 enrolled patients (45 IAPA patients and 82 CAPA patients). Aspergillus spp (culture) refers to culture-positive isolates that were not identified to the species level. **(A)** The absolute sample sizes of each Aspergillus species in IAPA and CAPA patients. **(B)** The relative proportions of IAPA and CAPA cases for each species.

*Aspergillus* cultures identified a total of 140 *Aspergillus* isolates. Among these, the following were identified: 85 *Aspergillus fumigatus* (*A. fumigatus*), 32 *Aspergillus flavus* (*A. flavus*), 14 *Aspergillus niger* (*A. niger*), 3 *Aspergillus terreus* (*A. terreus*), 2 *Aspergillus nidulans* (*A. nidulans*), 1 *Aspergillus versicolor* (*A. versicolor*), and 3 *Aspergillus* spp. (culture) as illustrated in Figure 2A. Among them, 13 patients (10.2%) had mixed infections with multiple *Aspergillus* species. *A. fumigatus* was the most frequently identified species among both IAPA and CAPA patients, accounting for 66.7 and 57.0%, respectively, no statistically significant difference was observed between the two groups, as detailed in Figure 2B.

### Clinical outcomes

It is noteworthy that 64 out of 82 CAPA patients received steroid treatment for SARS-CoV-2, resulting in an in-hospital mortality rate of 40.6% (26 out of 64). Conversely, the remaining 18 patients who did not receive steroids had a significantly lower in-hospital mortality rate of 22.2% (4 out of 18). Furthermore, a higher proportion of corticosteroid use was noted in the CAPA group compared to the IAPA group (*p* = 0.031). CAPA patients had a significantly longer interval from positive viral test to aspergillosis diagnosis compared to IAPA patients, with a median of 12.0 days (IQR: 8.0, 20.8) versus 2.0 days (IQR: 0.0, 6.0) (*p* < 0.001), as detailed in Table 3. Sensitivity results for patients after excluding individuals with prior immunosuppression are detailed in Supplementary Table S3.

**TABLE 3:** Clinical outcome of 82 CAPA patients and 45 IAPA patients.

Variables	Total (*n* = 127)	CAPA patients (*n* = 82)	IAPA patients (*n* = 45)	*p*-value
Antifungal treatment, n (%)	124(97.6)	82(100.0)	42(93.3)	0.079
Antiviral treatment, n (%)	119(93.7)	76(92.7)	43(95.6)	0.711
Mortality, n (%)	39(30.7)	30(36.6)	9(20.0)	0.053
In-put time median (IQR), days	18.0(11.0, 28.5)	20.0(11.0, 31.0)	17.0(12.0,21.0)	0.197
Corticosteroids use 7 days before and after ICU admission, n (%)	91(71.7)	64(78.0)	27(60.0)	**0.031**
Time from virus positive to aspergillus diagnosis, median (IQR), days	9.0 (3.0, 16.0)	12.0 (8.0, 20.8)	2.0 (0.0, 6.0)	**<0.001**
Respiratory failure at admission, n (%)	91(71.7)	64(78.0)	27(60.0)	**0.031**
MV, n (%)	74(58.3)	51(62.2)	23(51.1)	0.226
ICU admission, n (%)	68(53.5)	45(54.9)	23(51.1)	0.684
ICU mortality, n (%)	24(18.9)	18(22.0)	6(13.3)	0.235

Data are presented as number (%) or median (IQR) unless otherwise indicated. Bold indicated data with a significant difference.

Additionally, respiratory co-infections (bacterial) were assessed among the patients, revealing that 86 out of 127 patients (67.7%) tested positive for bacterial infections. Moreover, a statistically significant difference was observed in respiratory co-infections (bacterial) between IAPA and CAPA patients (χ^2^ = 4.715; *p* = 0.030). A total of 136 unique clinical strains were isolated from 127 patients, which included *Enterobacteriales* (*n* = 37), *Acinetobacter* spp. (*n* = 31), *Pseudomonas* spp. (*n* = 32), *Staphylococcus* spp. (*n* = 11), and other bacteria (*n* = 25) as detailed in Table 4. Sensitivity results for patients after excluding individuals with prior immunosuppression are detailed in Supplementary Table S4.

**TABLE 4:** Respiratory co-infections (bacterial) of 82 CAPA patients and 45 IAPA patients.

Variables	Total (*n* = 127)	CAPA patients (*n* = 82)	IAPA patients (*n* = 45)	*p*-value
Respiratory co-infections (bacterial), n (%)	86(67.7)	61(74.4)	25(55.6)	**0.030**
Total isolate strains, n	136	97	39	
*Enterobacteriales*, n (%)	37(27.2)	28(28.9)	9(23.1)	0.493
*Acinetobacter* spp., n (%)	31(22.8)	21(21.6)	10(25.6)	0.616
*Pseudomonas* spp., n (%)	32(23.5)	23(23.7)	9(23.1)	0.937
*Staphylococcus* spp., n (%)	11(8.1)	7(7.2)	4(10.3)	0.810
Others, n (%)	25(18.4)	18(18.6)	7(17.9)	0.934

Data are presented as number (%) or median (IQR) unless otherwise indicated. Bold indicated data with a significant difference. IAPA, influenza-associated pulmonary aspergillosis; COVID-19, coronavirus disease-19; CAPA, COVID-19-associated pulmonary aspergillosis.

**FIGURE 3:**
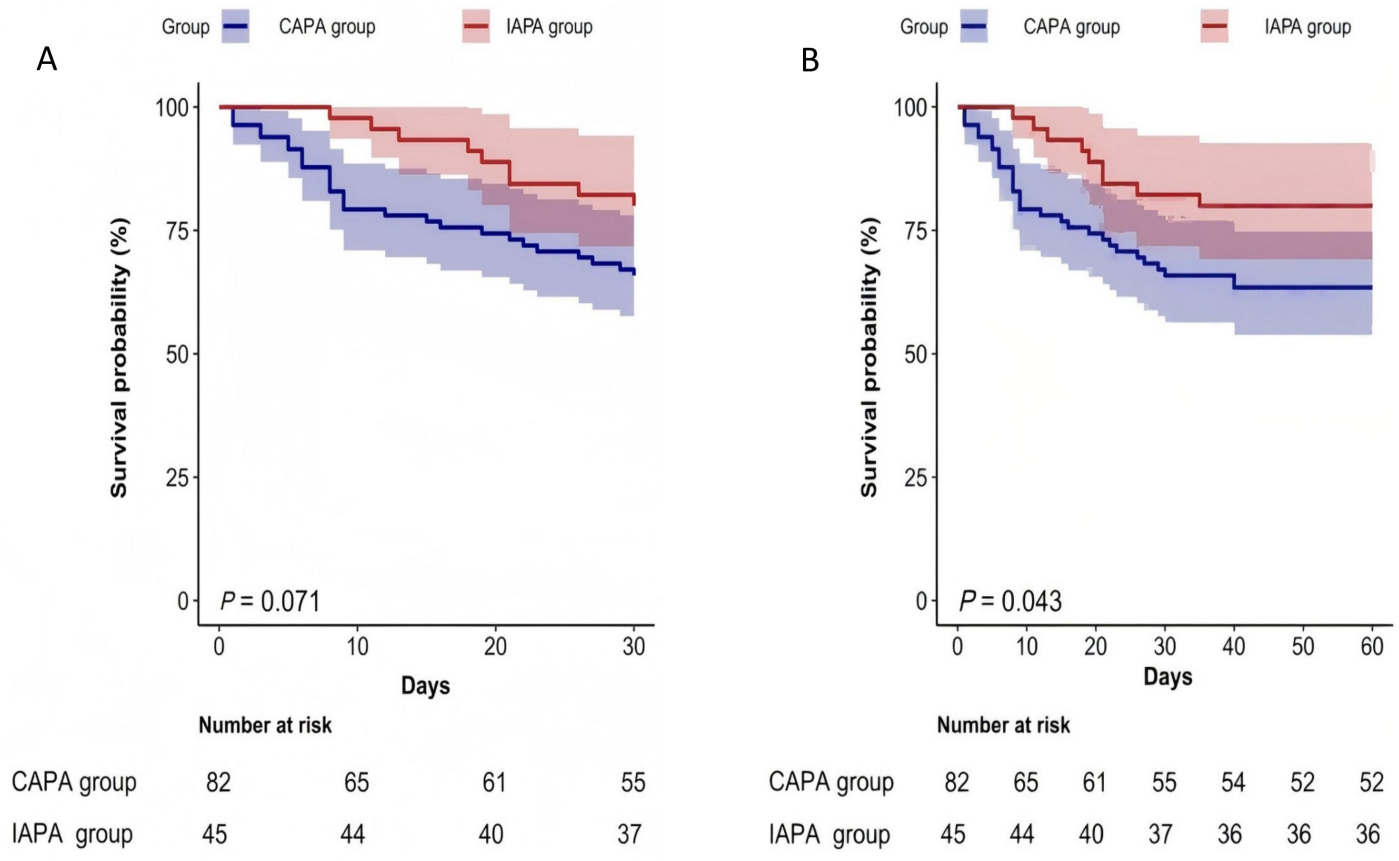
Kaplan-Meier survival curves for 30-day and 60-day mortality rates among CAPA and IAPA patients **(A,B)**. The x-axis of the Kaplan-Meier curves defines day 0 as the date of IPA diagnosis.

Survival analysis conducted 30 days post-diagnosis indicated a trend toward higher mortality in CAPA patients, though this did not reach statistical significance (*p* = 0.071); however, by 60 days, CAPA patients demonstrated a significantly increased mortality rate compared to IAPA patients (*p* = 0.043), as shown in Figure 3. Time-dependent Cox regression stratified into intervals of 0–14 days, 14–28 days, and 28–60 days, was performed. As shown in the Table 5, after adjusting for variables such as age, sex, lymphocytes, chronic pulmonary disease, and immunocompromised status, the analysis revealed a significant time-dependent effect on prognosis. During the first 14 days following IPA diagnosis, patients in the CAPA group had a significantly higher risk of mortality compared to those in the IAPA group (HR = 4.92, 95% CI: 1.35–18.01, *p* = 0.016). However, no statistically significant difference in mortality risk was observed between the two groups during the subsequent time intervals.

**TABLE 5:** Time-dependent Cox regression analysis for 60-day mortality (CAPA vs. IAPA).

Variables	0–14 days		14–28 days		28–60 days	
	HR (95% CI)	*p*-value	HR (95% CI)	*p*-value	HR (95% CI)	*p*-value
Model 1	3.70(1.09–12.57)	**0.036**	1.05(0.34–3.20)	0.937	2.69(0.30–24.08)	0.376
Model 2	3.48(1.01–12.02)	**0.049**	0.98(0.32–3.04)	0.978	2.53(0.28–22.73)	0.407
Model 3	4.92(1.35–18.01)	**0.016**	1.51(0.46–5.00)	0.498	3.26(0.35–30.25)	0.298

Model 1, not adjusted for confounders; Model 2, Model 1 + (age + sex); Model 3 = Model 2 + (lymphocytes + chronic pulmonary disease + immunocompromised status). Bold values indicate a statistically significant difference (*P* < 0.05).

## Discussion

This study explored the clinical characteristics of pulmonary aspergillosis in patients suffering from severe influenza and COVID-19, unveiling critical distinctions in the timing and risk factors linked to fungal infections. Our findings show that CAPA patients have a higher frequency of corticosteroid use and a significantly increased mortality rate compared to IAPA patients. Furthermore, CAPA patients exhibited a higher incidence of respiratory co-infections, including bacterial pathogens, underscoring the complex nature of these infections and the necessity for proactive screening and management strategies in critically ill individuals.

Our study showed that compared with IAPA patients, CAPA patients had a significantly longer median interval from positive viral test to aspergillosis diagnosis (12 days vs. 2 days). This finding aligns with the results reported by Reizine et al. (24), who observed delays in both diagnosis and antifungal initiation in CAPA relative to IAPA. The temporal disparity may be explained by differences in disease course: IAPA typically arises during the acute phase of viral pneumonia, whereas CAPA tends to occur in the plateau or late (immunoparalysis) phase, following sequential insults such as direct viral injury, immune dysregulation, and corticosteroid exposure (23). Moreover, prior studies suggest that influenza impairs antifungal immunity more profoundly than COVID-19, with IAPA manifesting as early as 48 h before ICU admission and CAPA developing within 1 week after (20, 34).

The pathophysiology of IAPA and CAPA is a “perfect storm” involving the virus, the fungus, and host immunity. The virus creates an immunosuppressive environment permissive for *Aspergillus* invasion and growth by disrupting physical barriers and perturbing key antiviral (Interferon) and antifungal (phagocyte, Th17) immune defenses. Although the specific mechanistic details and their relative importance differ between influenza and COVID-19, a core vicious cycle of epithelial damage, immune paralysis, and immunopathology underpins the pathogenesis of both (35).

Moreover, our analysis reveals that corticosteroid usage is markedly higher among patients with CAPA in comparison to those with IAPA. Although corticosteroids are essential for alleviating severe inflammatory responses, corticosteroid use increases vulnerability to respiratory co-infections, including aspergillosis (23, 36). This heightened risk may be attributed to the immunosuppressive characteristics of corticosteroids, which can hinder the host’s ability to mount a robust antifungal immune response (5, 17). Therefore, clinicians must carefully weigh the benefits and risks of corticosteroid therapy in these patients. They should also consider alternative treatments to reduce the risk of fungal infections.

Patients with IAPA and CAPA often present with respiratory co-infections, caused by common pathogens (e.g., *S. pneumoniae, S. aureus*) frequently occurring alongside aspergillosis. Moreover, these patients commonly experience microbiota dysbiosis (especially of the gut microbiota), resulting from a combination of factors, including the viral infection itself and extensive antibiotic use (35). Such dysbiosis, particularly alterations in the gut mycobiota, influences systemic and pulmonary immune responses (e.g., Th17 responses) via the “gut-lung axis,” thereby exacerbating susceptibility to *Aspergillus* and perpetuating a vicious cycle (35). When focusing specifically on CAPA patients, the rate of respiratory co-infections (bacterial) is even higher, with a Mexican study reporting 46%, a considerable proportion of which involved multidrug-resistant Gram-negative bacteria (37). In our investigation, the incidence of respiratory co-infections (bacterial) was notably higher in CAPA patients, which may be attributed to a combination of factors including corticosteroid treatment, extended hospitalization, and the extensive use of broad-spectrum antibiotics. These interventions can disrupt the normal bacterial flora and induce microbiome alterations, thereby creating an environment conducive to opportunistic pathogens and further increasing susceptibility to respiratory co-infections (38–40).

The key difference in prognosis between IAPA and CAPA is highlighted by significantly higher mortality rates in CAPA patients, indicating important clinical implications supported by recent studies (20–22). A Chinese study showed higher in-hospital mortality for CAPA patients (20), and a multicenter study confirmed worse survival curves for them (22). This higher mortality may stem from factors like a greater proportion of immunocompromised patients in the CAPA group, longer antibiotic and corticosteroid treatments, which can weaken antifungal defenses and increase infection risks (22, 24). Research indicates a non-linear relationship between corticosteroid use and mortality risk, particularly with prolonged treatment and high cumulative doses, suggesting differing immune responses in COVID-19 patients may complicate outcomes (41). Differing immune responses can cause hyperinflammation, affecting prognosis in respiratory co-infections (bacterial). CAPA linked to high mortality rates due to severe respiratory co-infections (37, 38). This study shows CAPA patients have a significantly higher 60-day mortality than IAPA patients, with most excess risk in the first 14 days (HR = 4.92). This indicates key differences in their clinical courses. First, the underlying pathophysiology and co-infection contexts differ. CAPA often occurs in the setting of severe pneumonia caused by SARS-CoV-2 infection, a virus that itself can induce significant lymphopenia and immune dysregulation (28). Furthermore, patients with COVID-19 are at high risk for fungal infections due to prolonged hospitalization and exposure to corticosteroids and broad-spectrum antibiotics (27, 29). Therefore, the high early mortality in CAPA highlights the critical need for early IPA detection and prompt antifungal therapy in severe COVID-19 cases.

A systematic review of studies on viral-associated pulmonary aspergillosis reported that the pooled *Aspergillus* culture positivity rate in BALF was 67% (range 63–89%) in influenza patients and 47% (range 41–53%) in COVID-19 patients (42). These findings provide a broader context for interpreting our results. Given the relatively high culture positivity rate observed, we offer the following explanations. First, not all patients in our cohort underwent bronchoscopy; BALF sampling was performed predominantly in patients admitted to the ICU, particularly those requiring mechanical ventilation. Second, patients selected for bronchoscopic sampling in our study tended to have more severe disease, with more typical clinical presentations or radiological findings suggestive of IPA. Third, in this study, repeated BALF sampling was performed in some patients, which may have contributed to the higher observed positivity rate. These factors likely contributed to higher pulmonary fungal burdens and an increased likelihood of positive BALF cultures.

Nonetheless, our study had several limitations. It was a single-center study with a limited sample size, and not all patients underwent bronchoscopy or BALF GM testing. Additionally, due to institutional constraints, PCR testing of BALF was not performed, which to some extent affected diagnostic specificity. The retrospective design led to missing detailed imaging reports and bronchoscopy records for some patients, potentially resulting in an underestimation of the number of patients meeting the diagnostic criteria. Furthermore, patient enrollment was concentrated during the Omicron wave, which may limit the applicability of our findings to other viral variants. Although the inclusion of a small subset of immunocompromised patients reflects real-world clinical populations in tertiary referral centers, it also constitutes a limitation. Future prospective studies should incorporate standardized imaging, endoscopic examinations, and PCR testing to enhance diagnostic sensitivity and specificity.

## Conclusion

CAPA patients had a significantly longer median interval from positive viral test to aspergillosis diagnosis. CAPA was independently associated with a nearly fivefold increase in the risk of death within the first 14 days following IPA diagnosis. This time-dependent hazard, together with the higher frequency of corticosteroid use, respiratory co-infections (bacterial), and severe lymphopenia, highlight the multifactorial nature of CAPA pathogenesis and emphasize the need for integrated management strategies in critically ill patients. Further studies are warranted to elucidate underlying mechanisms and to establish optimized preventive and therapeutic approaches.

## Data Availability

The original contributions presented in the study are included in the article/Supplementary material, further inquiries can be directed to the corresponding author.
