# Supplementary material for: Comparative clinical features and outcomes of invasive pulmonary aspergillosis following influenza versus COVID-19: a retrospective cohort study
Source: Front Med (Lausanne). 2026 Apr 24;13:1790007. doi: 10.3389/fmed.2026.1790007 (PMC13153091; doi:10.3389/fmed.2026.1790007)
Supplement: Supplementary file 1 [file Table_1.pdf]

Supplementary Materials

Explicitly reported the number of patients with pre-existing immunocompromising conditions in each group:

IAPA group: There are 41 immunocompetent patients and 4 Immunocompromised patients: There were 2 cases of hematological malignancies, 8 cases of autoimmune diseases, and 1 case of solid organ transplantation. Among them, 2 patients with hematological malignancies also had autoimmune diseases (1 with connective tissue disease and 1 with Sjögren's syndrome), and 1 patient with solid organ transplantation (lung transplant) had a hematological malignancy. Therefore, after excluding duplicates, there were 6 patients with autoimmune diseases alone (2 with connective tissue disease, 3 with rheumatoid arthritis, and 1 with Sjögren's syndrome). In total, the IAPA group had 4 patients with underlying immunocompromising conditions (2 patients with hematological malignancies and 2 patients with connective tissue disease).

CAPA group: There are 69 immunocompetent patients and 13 immunocompromised patients: There were 7 cases of hematological malignancies, 7 cases of autoimmune diseases, and 6 cases of organ transplantation. Among these, 3 patients with hematological malignancies also had autoimmune diseases (2 with connective tissue disease and 1 with Sjögren's syndrome), and 2 patients with hematological malignancies had a history of solid organ transplantation (1 lung transplant and 1 kidney transplant). Therefore, after excluding duplicates, there were 2 patients with hematological malignancies alone, 4 patients with autoimmune diseases alone (2 with ANCA-associated vasculitis and 2 with rheumatoid arthritis), and 4 patients with solid organ transplantation alone (2 kidney transplants, 1 liver transplant, and 1 heart transplant). In total, the CAPA group had 13 patients with underlying immunocompromising conditions (7 patients with hematological malignancies, 4 with solid organ transplantation, and 2 with connective tissue disease).

Table S1. Baseline features of the study patients without immunosuppression

Variables	Total (n=110)	CAPA patients (n=69)	IAPA patients (n=41)	*p*-value
**Demographics**				
Male, n (%)	72 (65.5)	49 (71.0)	23 (56.1)	0.112
Age, Mean ± SD, years	68.1±13.4	70.3±12.5	64.2±14.1	**0.020**
BMI, Mean ± SD, kg/m^2	23.5 ± 3.8	23.6± 4.1	23.3 ± 3.2	0.732
**Underlying conditions,** n **(%)**				
**Arterial** Hypertension	54 (49.1)	34 (49.3)	20 (48.8)	0.960
Coronary artery disease	50 (45.5)	35 (50.7)	15 (36.6)	0.150
Cerebrovascular disease	12 (10.9)	7 (10.1)	5 (12.2)	0.759
Diabetes mellitus	44 (40.0)	25 (36.2)	19 (46.3)	0.295
Chronic kidney disease	10 (9.1)	8 (11.6)	2 (4.9)	0.316
Chronic pulmonary disease	55 (50.0)	26 (37.7)	29 (70.7)	**<0.001**
Chronic liver disease	8 (7.3)	7 (10.1)	1 (2.4)	0.254
Hematological malignancy	10 (9.1)	5 (7.2)	5 (12.2)	0.496
Solid organ transplantation	16 (14.5)	11 (15.9)	5 (12.2)	0.590
Vaccine	29(26.4)	17(24.6)	12(29.3)	0.594
**Symptoms,** n (%)				
Fever	96 (87.3)	64 (92.8)	32 (78.0)	**0.025**
Cough	100 (90.9)	62 (89.9)	38 (92.7)	0.741
Hemoptysis	18 (16.4)	10 (14.5)	8 (19.5)	0.491
Diarrhea	10 (9.1)	9 (13.0)	1 (2.4)	0.087
Vomit	6 (5.5)	6 (8.7)	0(0)	0.082
Sore throat	18 (16.4)	12 (17.4)	6 (14.6)	0.705
**Laboratory tests at admission**				
Leukocyte count, median (IQR), ×10^9/L	8.7(5.6, 11.8)	9.1 (5.8, 12.0)	7.9 (5.6, 10.6)	0.491
Neutrophils, median (IQR), ×10^9/L	7.1 (4.5, 10.7)	7.7 (5.1, 11.2)	6.2(4.2, 9.3)	0.292
Lymphocytes, median (IQR), ×10^9/L	0.6(0.3, 1.2)	0.5(0.3, 0.9)	0.8(0.6, 1.6)	**<0.001**
Hemoglobin, median (IQR), g/L	123.8±21.0	123.3±22.7	124.6±18.2	0.755
CRP, median (IQR), mg/L	48.6 (18.0, 106.0)	59.5 (13.0, 107.0)	47.5 (22.0, 100.0)	0.589
Creatinine, median (IQR), μmol/L	62.2 (52.4, 79.1)	62.9 (52.4, 78.1)	61.4 (51.3, 80.0)	0.703

PCT, median (IQR), ng/mL	0.1 (0.0, 0.8)	0.1 (0.0, 0.8)	0.1(0.0, 0.8)	0.890
$CD4^{+}$Tcells, median (IQR), cell/μL	173.5 (80.0, 354.2)	140.0 (75.0 , 286.0)	257.0(137.0, 661.0)	**0.005**
$CD8^{+}$ T cells, median (IQR), cell/μL	108.5(57.8, 259.0)	101.0(44.0, 183.0)	183.0 (86.0, 369.0)	**0.016**
NK cells, median (IQR), cell/μL	60.5 (33.5, 139.0)	61.0 (35.0, 129.0)	59.0 (33.0, 212.0)	0.469
B cells, median (IQR), cell/μL	81.5 (24.5, 156.2)	67.0(19.0, 133.0)	98.0(26.0, 170.0)	0.254
D-Dimer, median (IQR), ng/L	1.2 (0.6, 2.4)	1.2(0.8, 2.9)	1.0(0.5, 1.8)	0.221

Notes: Data are presented as number (%) or median (IQR) unless otherwise indicated. Bold indicated data with a significant difference

Abbreviations: BMI, body mass index; CRP, C-reaction protein; PCT, procalcitonin; NK, natural killer cell; MV, mechanical ventilation.

Table S2. Laboratory diagnositics of *Aspergillus* of the 110 enrolled patients

Variables	N	CAPA patients (n=69)	IAPA patients (n=41)	*p*-value
BALF culture, n	84	55	29	
BALF culture (+), n (%)	59(70.2)	36(65.5)	23(79.3)	0.187
Sputum culture, n	47	27	20	
Sputum culture (+), n (%)	13(27.7)	4(14.8)	9(45.0)	**0.022**
Mircoscopy, n	43	30	13	
Mircoscopy (+), n (%)	9(20.9)	4(13.3)	5(38.5)	0.063
Serum GM, n	100	68	32	
Serum GM (+), n (%)	40(40.0)	24(35.3)	12(37.5)	0.830
BALF GM, n	36	22	14	
BALF GM (+), n (%)	28(77.8)	18(81.8)	10(71.4)	0.465

Notes: Data are presented as number (%). Bold indicated data with a significant difference.There were cases of repeated sample submissions per patient; however, to maintain statistical independence and avoid inflation of the incidence rate, only the results of the first culture from each patient were included in the final statistical analysis

Table S3. Clinical outcome of 69 CAPA patients and 41 IAPA patients

**Variables**	**Total (n=110)**	**CAPA patients (n=69)**	**IAPA patients (n=41)**	***p*-value**
Antifungal treatment, n (%)	107(97.3)	69(100.0)	38(92.7)	**0.023**
Antiviral treatment, n (%)	102(92.7)	63(91.3)	39(95.1)	0.708
Mortality, n (%)	32(29.1)	23(33.3)	9(22.0)	0.334
In-put time median (IQR), days	18.0(12.0, 30.0)	20.0(12.0, 33.0)	17.0(12.0, 22.0)	0.184
Corticosteroids use 7 days before and after ICU admission, n (%)	78(70.9)	54(78.3)	24(58.5)	**0.028**
Time from virus positive to aspergillus diagnosis, median (IQR), days	9.0 (3.2, 15.8)	12.0 (8.0, 20.0)	3.0 (0.0, 7.0)	**<0.001**
Respiratory failure at admission, n (%)	76(69.1)	53(76.8)	23(56.1)	**0.023**
Mechanical ventilation, n (%)	66(60.0)	46(66.7)	20(48.8)	0.064
ICU admission, n (%)	62(56.4)	41(59.4)	21(51.2)	0.402
ICU mortality, n (%)	20(18.2)	14(20.3)	5(12.2)	0.277

Notes: Data are presented as number (%) or median (IQR) unless otherwise indicated. Bold indicated data with a significant difference.

Table S4. Respiratory co-infections(bacterial) **of 69 CAPA patients and 41 IAPA patients**

Variables	Total (n=110)	CAPA patients (n=69)	IAPA patients (n=41)	*p*-value
Respiratory co-infections, n (%)	73(66.4)	51(73.9)	22(53.7)	**0.030**
Total Isolate strains, n	100	69	31	
Enterobacteriales, n (%)	27(27.0)	20(29.0)	7(22.6)	0.505
Acinetobacter spp, n (%)	24(24.0)	15(21.7)	9(29.0)	0.430
Pseudomonas spp, n (%)	25(25.0)	18(26.1)	7(22.6)	0.708
Staphylococcus spp, n (%)	6(6.0)	4(5.8)	2(6.5)	1.000
Others, n (%)	18(18.0)	12(17.4)	6(19.4)	0.813

Notes: Data are presented as number (%) or median (IQR) unless otherwise indicated. Bold indicated data with a significant difference.

Abbreviations: IAPA, influenza-associated pulmonary aspergillosis; COVID-19, coronavirus disease-19; CAPA, COVID-19-associated pulmonary aspergillosis.
